# Antibody–drug conjugates in cancer and beyond: progress, promise, and perspectives

**DOI:** 10.1093/abt/tbaf013

**Published:** 2025-06-11

**Authors:** Victor S Goldmacher

**Affiliations:** Research and Development Department, ImmuVia, Inc., Cambridge, MA 02142, United States

**Keywords:** antibody–drug conjugate, ADC, targeted therapy, antibody

## Abstract

Antibody–drug conjugates (ADCs) are transforming cancer therapy by combining antibody specificity with potent cytotoxic agents, enabling targeted tumor cell killing while minimizing systemic toxicity. This special collection of Antibody Therapeutics presents a wide overview of recent advances in ADC research and development. Topics include targeting strategies, antibody formats, innovative payloads and bispecific apoptosis triggers, formulation strategies, toxicity profiling, and conjugation technologies. Together, these contributions reflect the rapid evolution of the ADC field and point toward safer, more effective therapies for cancer and beyond.

## Teaser

**Statement of Significance** This special collection highlights recent advances in antibody–drug conjugates—spanning targeting strategies, formats, payloads, bispecific apoptosis triggers, formulations, toxicity, and conjugation—reflecting the field’s rapid evolution toward safer, more effective cancer therapies.

Antibody–drug conjugates (ADCs) combine the target specificity of monoclonal antibodies with the cytotoxic potency of small-molecule drugs, aiming to reduce systemic toxicity by delivering the payload preferentially to tumor cells. As the technologies underpinning ADC design and manufacturing have matured, the field has expanded significantly, with a growing number of ADCs approved for clinical use and many more in late-stage development. The diversification of tumor-associated targets and cytotoxic payloads is broadening the spectrum of cancer indications amenable to ADC therapy. In parallel, the emergence of novel antibody formats and payloads that engage the tumor microenvironment promises to enhance intratumoral drug distribution and efficacy, particularly in resistant or poorly vascularized tumors. Notably, the utility of ADCs is also beginning to extend beyond oncology, opening new avenues for therapeutic applications.

This special issue highlights recent advances in ADC research and development. The collection of articles and reviews offers a broad perspective on the progress, innovation, and ongoing challenges in harnessing ADCs for cancer and beyond.

Ma and colleagues at Fudan University presented a detailed review of the emerging potential of smaller-format ADCs [1]. Conventional ADCs employing full-length immunoglobulin G (IgG) antibodies face challenges in penetrating solid tumors due to their large size. To overcome this limitation, miniaturized antibody fragments with engineered formats have been explored for ADC development. Owing to their smaller molecular size, these antibody fragment–drug conjugates offer improved tumor penetration and enhanced delivery of cytotoxic payloads, potentially resulting in superior therapeutic outcomes. The review highlights recent advances in ADCs based on smaller antibody formats with particular emphasis on nanobodies, other single-domain antibodies, and non-antibody scaffold proteins such as fibronectins, DARPins, and affibodies, which are increasingly being applied in novel ADC designs.

Several Human Epidermal Growth Factor Receptor 2 (HER2)-targeting ADCs have received regulatory approval for the treatment of HER2-expressing metastatic cancers. Among the most notable HER2-targeting ADCs are Kadcyla (developed by Genentech) and DS-8201a (trastuzumab deruxtecan), a novel HER2-targeting ADC carrying a DNA topoisomerase I inhibitor. However, current ADCs still face limitations, including drug resistance and toxicity associated with their payloads. Eribulin, approved for the treatment of metastatic breast cancer and liposarcoma, has emerged as a promising payload with a distinct mechanism and potentially improved safety. To explore this, Wang *et al*. developed BB-1701, a novel HER2-targeting ADC conjugated with eribulin [2], which is currently being evaluated in a Phase 1 clinical trial. BB-1701 employs eribulin, a microtubule dynamics inhibitor with a mechanism of action distinct from that of the maytansinoids used in Kadcyla (ado-trastuzumab emtansine) and the auristatins used in several other HER2-targeting ADCs. In contrast to these established payloads, eribulin offers a unique pharmacological profile. The ADC demonstrated potent cytotoxicity, including in HER2-low cell lines, where it outperformed HER2-targeting ADCs conjugated to DM1, a derivative of maytansine, a microtubule dynamics inhibitor, or Dxd, a topoisomerase I inhibitor. BB-1701 also showed robust activity in tumor models resistant to DM1- or Dxd-containing ADCs.

Ye and colleagues at Xiamen University present a new strategy for the treatment of chronic hepatitis B, addressing the persistent challenge posed by immune evasion and liver-specific immunosuppression in human papillomavirus (HBV) infection [3]. The study introduces an immune-stimulating antibody conjugate that combines the antiviral specificity of an HBV-neutralizing antibody with the immune-activating properties of a Toll-like receptor 7/8 (TLR7/8) agonist. These two components complement each other: broadly neutralizing antibodies provide targeted antiviral activity and immune activation, while TLR7/8 agonists help overcome immune tolerance—when combined as 129G1-IMDQ, they aim to enhance efficacy while minimizing systemic toxicity. In a preclinical model using adeno-associated virus (AAV)/HBV-infected mice, short-term administration of 129G1-IMDQ led significantly reduced circulating hepatitis B surface antigen (HBsAg) levels.

Chang and colleagues at BioAtla Inc. reported on the preclinical development of mecbotamab vedotin (BA3011), an a cell surface receptor tyrosine kinase (AXL)-targeted ADC [4]. AXL, a receptor tyrosine kinase, is overexpressed in numerous solid and hematologic malignancies and is associated with disease progression, poor prognosis, and resistance to therapies—particularly tyrosine kinase inhibitors—via upregulated AXL signaling or pathway switching. Early therapeutic approaches using naked antibodies were unsuccessful due to the high doses required for efficacy, while conventional AXL-targeting ADCs suffered from dose-limiting toxicities driven by AXL expression in normal tissues. To overcome these challenges, BioAtla developed a conditionally active ADC designed to bind AXL preferentially in the acidic tumor microenvironment. Its binding affinity to AXL is significantly higher at pH 6.0 than at physiological pH 7.4, thereby minimizing off-tumor effects in healthy tissues. BA3011 demonstrated tumor-specific binding and cytotoxicity against AXL-positive cancer cell lines in vitro. In xenograft models, mecbotamab vedotin showed strong antitumor activity. Beyond preclinical studies, BA3011 has also shown preliminary signs of clinical activity at tolerable doses in patients with advanced solid tumors.

Our group (Goldmacher *et al*. [5]) reviewed the current state of monospecific apoptosis inducers targeting death receptor 4 and/or death receptor 5 (DR5), as well as the emerging class of bispecific apoptosis triggers (BATs)—bispecific antibodies designed to simultaneously engage a tumor-associated antigen and a death receptor on cancer cells, thereby directly activating the extrinsic apoptotic pathway. Unlike ADCs, which rely on the delivery of cytotoxic payloads, BATs employ an anti-death receptor component to trigger apoptosis without harming healthy tissues. Although the field is still in its early stages, recent advancements are highly encouraging. Analysis of preclinical and clinical data for DR5-targeting antibodies has been instrumental in defining the criteria for next-generation therapies that are both effective and safe. BATs have the potential to overcome limitations associated with ADCs, reach broader patient populations, and reduce systemic toxicity. Following our review, we introduced Cancerlysin™ IMV-M, a first-in-class BAT that demonstrated potent efficacy across diverse xenograft cancer models and favorable safety in non-human primates, representing a significant step forward in the development of targeted cancer therapeutics [6].

Systemic toxicity remains a critical factor limiting the maximum tolerated dose and therapeutic index of ADCs. Cheng and collaborators reviewed the toxicity profiles of various approved ADCs, drawing from both preclinical and clinical data [7]. Their analysis highlights how the structural components of ADCs—including the payload (ranging from small molecules to radionuclides such as ^90^Y and ^131^I, toxic proteins like PE38, photoabsorbers like IRDye700DX, and oligonucleotides), drug-to-antibody ratio, linker chemistry, target antigen, antibody affinity and biophysical properties, Fcγ receptor and FcRn interactions, and conjugation technologies—impact toxicity. The review covers diverse toxicities, including hematologic, gastrointestinal, skin, peripheral neuropathy, ocular, hepatic, and pulmonary toxicities, with a particular emphasis on interstitial lung disease. This review provides valuable insights that will inform the design and development of next-generation ADCs with improved safety profiles.

Wen *et al*. reviewed the formulation strategies of FDA-approved ADCs, offering valuable insights for the development of new ADC formulations [8]. Notably, 14 out of 16 FDA-approved ADCs are formulated as lyophilized products, reflecting the general perception that ADCs are less stable than unmodified antibodies. The development of ADC formulations—particularly liquid formulations—presents unique challenges due to their complex structures, diverse physicochemical properties, and multiple degradation pathways. This review outlines the technical challenges specific to ADCs compared to unconjugated antibodies and discusses corresponding solutions. It provides comprehensive analysis of formulation strategies employed across commercial ADCs and highlights key considerations throughout the formulation development process, from early-stage formulation to final drug product manufacturing. The authors also offer strategic perspectives on future directions to advance this rapidly evolving therapeutic class.

Fan and colleagues present a comprehensive review of conjugation technologies for ADCs [9]. The authors compare the most prevalent and promising conjugation strategies used in clinical ADC pipelines, evaluating their characteristics, advantages and limitations, Chemistry, Manufacturing, and Controls (CMC) potential, and clinical status. These include random lysine conjugation and a range of site-specific approaches: interchain cysteine conjugation (highlighting advances from WuXi, Abzena, Pfizer, and others), enzymatic-tag conjugation, glycan remodeling, affinity peptide conjugation, engineered cysteine insertion, and incorporation of non-natural amino acids. Identifying optimal conjugation sites and chemistries remains challenging, as these choices significantly influence binding, internalization, payload release, pharmacokinetics, and effector functions. While newer technologies often offer improved efficacy and safety, they may lack clinical validation and present unexpected CMC hurdles. Consequently, some developers opt for established methods when their programs already carry risks from novel targets or payloads.

The field of ADCs is expanding rapidly and has emerged as one of the fastest-growing classes of anti-cancer therapeutics, with global annual sales exceeding $10 billion in 2023 and projected to rise sharply. This makes it a timely moment to highlight recent advances through this special collection in Antibody Therapeutics. The original articles and reviews included here explore key aspects of the evolving ADC landscape—though not all, as the field has grown too broad to capture comprehensively in a single volume. Collectively, these contributions showcase the expanding ADC toolbox aimed at improving the therapeutic index. We anticipate continued innovation in this dynamic field, with future advances bringing us ever closer to more powerful and safer cancer therapies.

## Data Availability

Not applicable.
